# The Impact of COVID-19 Restrictions on Mental Well-Being and Working Life among Faroese Employees

**DOI:** 10.3390/ijerph18094775

**Published:** 2021-04-29

**Authors:** Annika H. Davidsen, Maria S. Petersen

**Affiliations:** Department of Occupational Medicine and Public Health, The Faroese Hospital System, 100 Torshavn, Faroe Islands; maria@health.fo

**Keywords:** COVID-19, employee wellbeing, working environment, working parents, Faroe Islands

## Abstract

The societal changes caused by COVID-19 have been far-reaching, causing challenges for employees around the world. The aim of this study was to assess the effect of the COVID-19 restrictions on mental well-being, working life, family life and social life among Faroese employees within a broad range of professions. A total of 1328 Faroese employees answered an anonymous self-report survey from 13 April to 4 May 2020. Employee mental well-being was only modestly affected by the restrictions and the respondents had a mean score of 50.7 on the Warwick–Edinburgh Mental Wellbeing Scale where a score between 41–44 is found to correspond with possible depression. Work commitment, work and family life, work satisfaction and work ability were all rated significantly worse after the COVID-19 outbreak than before (all *p* values < 0.005). Contrary to previous research, employees in health services assessed their work ability significantly higher than employees in teaching, and child and youth care (*p* < 0.05). Working parents had higher levels of stress and assessed their work ability significantly lower than employees without children (*p* < 0.05), and women tended to be more worried than men because of the pandemic. In conclusion, the overall mental well-being of Faroese employees was on an average level during lock-down in April and May 2020. Their working life seemed, however, to be worse than usual.

## 1. Introduction

The societal changes caused by COVID-19 (Corona Virus Disease-19) have been far-reaching, as we face the second and third waves around the world. The strong measures taken to limit the spread of the disease have caused challenges for employees, and, since March 2020, there has been a large shift in the way we work [1,2]. Most professional branches have been forced to solve their work-related tasks differently than usual: Working from home, working alone, working in small groups, etc., which, among other things, has made it more difficult to stay in touch with managers and colleagues.

The distinction between ‘essential’ and ‘non-essential’ functions [1] was early in the first phase a part of the official guidelines. The essential functions such as grocery stores, the acute functions in the hospital system, care homes for elderly and disabled people and child care for employees in the essential functions were to stay open, whereas the non-essential functions such as beauty parlors, child care for non-essential staff and schools were to close their services or let their employees solve their tasks remotely. These sudden changes have made the topic of employee well-being more critical and may pose different challenges for different groups of employees. For employees in the essential functions, the infection risk might lead to mental health concerns, stress and anxiety [2,3], while employees working remotely are at risk for experiencing loneliness and isolation, which has been associated with depression, suicidal behavior and other mental difficulties [4]. One study found perceived stress among individuals working from home to increase during COVID-19 [5].

The importance of strong and positive relationships in workplaces, also referred to as social capital [6], might in these times be even more relevant and difficult to maintain. Working from home, working alone, working in small groups and other ways of re-organizing team work may compromise the bonding social capital, which is based on close workplace relations, such as team work and other group work, where direct and frequent interaction is needed to solve the work-related tasks [6]. Workplace social capital has in previous research been linked to employee wellbeing and engagement [6,7,8].

Only few studies have been published about the impact of COVID-19 restrictions on mental well-being and working environment among employees, and most have targeted healthcare professionals or frontline professionals. In a literature review, Domenicano found that healthcare workers reported high levels of stress, anxiety and severe symptoms of depression [9]. Accordingly, Wanigasooriya and colleagues found a high prevalence of clinically significant symptoms of anxiety, depression and post-traumatic stress disorder (PTSD) among British hospital workers [10]. In Lai and colleagues’ study [11], Chinese health care workers reported symptoms of depression, anxiety, insomnia and distress, whereas Evanoff and colleagues reported stress, anxiety, depression, work exhaustion and burnout among American university staff [12]. In a survey among the general Italian population, respondents reported scores within clinical ranges for overall distress, depression, anxiety and PTSD [13]. Similarly, a cross-sectional population study in Spain showed that around 30% of the respondents exhibited symptoms within the clinical range of depression, anxiety and stress [14] Unsafe and poor working conditions of health-care workers, especially in the long-term care sector, makes residents and employees more vulnerable to COVID-19 infection, morbidity and mortality [15]. In some countries, nursing homes have been severely affected by the pandemic; in May 2020, 53.1% of all COVID-19 related deaths in Belgium and 51% of all COVID-19 related deaths in France occurred in nursing homes [16]. Overall, these results indicate that employees can be severely affected by COVID-19; especially health care employees are at risk for developing unfavorable mental health and mental health problems.

Other studies have looked at the consequences of COVID-19 on family life and working parents. Many parents are, under normal circumstances, dissatisfied with the balance between work and family life [17], but home-schooling and lack of childcare during COVID-19 does seem to pose a challenge for some working parents, making family life more stressful than under normal circumstances [18]. A limited number of studies have been published around this theme. However, one study found that having young children at home was significantly associated with mood-worsening during lock-down [19]. In another survey among 3013 Americans, parents with children under the age of 18 reported a significantly higher stress level compared to adults without children [20]. With children out of school, increased care for the elderly, etc., authors have voiced a concern about the increased care burden of women during COVID-19 [21]. Short-term effects of daily stressors on family dynamics can have cumulative, long-term implications for family health and functioning [22], which is one argument for the importance of the mental well-being of the whole family.

The Faroe Islands consist of 18 islands, situated in the North Atlantic between Great Britain, Iceland and Norway. In March 2021, the Faroes had around 53,000 inhabitants. The birth rate is high; in 2019, the average Faroese woman had 2.5 children. Most households are dual-earner households, although Faroese men typically work more paid hours than Faroese women. Of 26,828 employees in February 2021, 13,077 were women [23]. The first confirmed COVID-19 case in the Faroe Islands was announced on 3 March 2020. During the first phase (March–May 2020) of the COVID-19 outbreak in the Faroe Islands, several restrictions, all non-mandatory, applied in order to limit the spread of COVID-19 in the Faroe Islands: lock-down of schools, colleges and universities meant that students of all ages either participated in online teaching or were home-schooled in an extent that varied from school to school. Many workplaces encouraged their employees to work from home when possible, some were closed because of lack of activities (e.g., branches connected to tourism), and other workplaces rearranged their activities, e.g., by dividing employees in groups and limiting interaction between groups of colleagues. Social distancing was recommended, and people were encouraged to avoid social gatherings. After the first case, other cases followed quickly causing a spike in March, another spike in August, and yet another spike in December 2020. The government´s approach has from the beginning been large scale testing, contact tracing and isolation combined with social distancing. Testing is still mandatory for visitors to the country [24,25]. At the time of writing, the Faroes have recorded 661 cases of COVID-19 and there has been one fatality from COVID-19 in the Faroe Islands [25].

The aim of this study was to assess the effect of COVID-19 on mental well-being among Faroese employees within a broad range of professions. Because the pandemic put an immediate strain on the health care systems worldwide, we hypothesized that health care professionals’ mental well-being and experience of working environment during lock-down would be more negatively affected than the mental well-being of other professionals. We also hypothesized that working parents would experience poorer mental well-being and higher levels of stress during lock-down, because of home-schooling, the lack of childcare and a general shift in domestic care obligations.

## 2. Materials and Methods

### 2.1. Study Design and Participants

The anonymously completed self-report survey was available on the online platform SoSci Survey [26], from 13 April until 4 May 2020. The survey was distributed via social media and facilitated to employees through Faroese unions. They survey was open to all employees older than 18, with access to the internet. Participation was voluntary and data collection was maintained fully anonymous, e.g., by not collecting any data that could potentially identify a respondent. 

### 2.2. Survey Items

The survey consisted of 76 questions, targeted at examining how the COVID-19 restrictions affected the mental well-being, working life, family life and the social life of Faroese employees during lock-down. The questions in the survey also addressed how respondents experienced circumstances around their work situation (e.g., job satisfaction and perceived stress) under normal circumstances and during the COVID-19 outbreak. In addition, sociodemographic data were collected in the survey including age, sex, marital status, family situation, education and employment status, rating of overall health and possible mental health issues. The questionnaire was tailored by the authors to address the research questions. The rationale was to use multiple validated tools concerning mental well-being (Warwick–Edinburgh Well-being Scale) and working environment (from the Danish survey ‘Working Environment and Health’), and to maintain high feasibility.

The Warwick–Edinburgh Mental Well-being Scale (WEMWBS) was used to assess the respondents´ mental well-being the last 2 weeks. WEMWBS is a 14-item scale covering subjective well-being and psychological functioning, in which all items are worded positively and address aspects of positive mental health. The scale is scored by summing responses to each item answered on a 1 to 5 Likert scale. The minimum scale score is 14 and the maximum is 70. A high score indicates good mental well-being whereas a low score indicates poor mental well-being [27,28,29]. A score of 40 and below has been found to correspond to probable depression and a score of 41–44 to possible depression [30]. Population studies in Denmark, England and Catalonia found mean scores ranging between 49.8 and 58.1 [28]. A survey among Faroese high school students found a mean score of 50.9 (ranging from 49.0–55.5) [31].

The survey items addressing psychosocial working environment were translated to Faroese from the Danish survey ‘Arbejdsmiljø og Helbred’ (‘Working environment and Health’) developed by the Danish National Research Centre for the Working Environment [32]. The items addressed four aspects of working life: work commitment (five items: self-confidence; interesting tasks; importance of work; cheerful at work; preoccupied at work), work and family life (eight items: time at work; rate of working; deadlines; time pressure; availability after work; overtime; energy at work; work time vs leisure time), work satisfaction (1 item) and work ability (1 item). All but one item were answered on a 5-point Likert scale with answers ranging from ‘not at all’, ‘to a very little extent’, ‘to some extent’ ‘to a great extent’ to ‘a very great extent’ [33]. The respondents were asked to answer the same questions twice: (1) ‘as they usually would assess their job’ and (2) ‘as they would assess their job during COVID-19′.

The survey items ‘effects of COVID-19 on working life, family life and social life’ and ‘coping with changes caused by COVID-19′ had a free text option, where respondents could write other effects or other ways of coping than the answer options allowed.

### 2.3. Statistical Analysis

Data are presented as number of respondents and percentage (of total number of respondents) for categorical data and mean and standard deviation (SD) for normally distributed data. Paired samples *t*-tests were used to assess possible differences in working environment (work commitment, work and family life, work satisfaction and work ability) pre and during COVID-19. Binary logistic regression analysis was used to examine the relationship between good or poor mental well-being and these predictors: perceived stress, job satisfaction, work ability, age, education, gender and children. Binary logistic regression analysis was also used to examine the relationship between being an employee with children under the age of 18 or not and the same predictors. Multiple analysis of variance tests (ANOVAs) were used to explore possible differences between different professions in the survey concerning the items perceived stress, mental well-being, (during COVID-19) job satisfaction and (during COVID-19) work ability. The free text responses were analyzed by the first author, by reviewing all the comments and organizing them in categories.

Data were analyzed using the Statistical package for the Social Sciences (IBM SPSS v. 25, IBM, Armonk, NY, USA) [34].

## 3. Results

A total of 1328 people participated in the survey with a mean age of 34 (ranging from 19–71 years); 77% (*n* = 1025) were women. The majority (74.5%) of the respondents were employed in the public sector, and the largest branches were teaching, health services and jobs within child and youth care, such as daycare, kindergarten and recreation centers. Other respondents were employed in, e.g., social agencies, production industry, building industry, long-term care sector, financial sector and within administration. Nineteen percent were employed in the private sector. Most of the participants were married or in a relationship, and more than half of the participants had children under the age of 18 (Table 1).

### 3.1. The Relationship between COVID-19 Restrictions and Mental Well-Being and Perceived Stress

In total, 71% of the respondents evaluated their current overall health as good or very good. Furthermore, 79.2% of the respondents reported that their mental well-being, not at all or only to a small extent, was affected by the COVID-19 restrictions. The respondents’ mental well-being was also assessed in the WEMWBS scale and showed a mean score of 50.7 (SD = 8.1, median = 51.0). The percentage of respondents scoring below 44 (signaling depression), was 21.8. When asked about perceived stress the last 2 weeks, 12.6% had felt stressed, and the majority (42.1%) in this group pointed to the combination of work and family life as the greatest source of stress (as opposed to work or family life). Of the respondents, 34.4% answered that COVID-19, to a large or very large extent, had caused concerns. Table 2 illustrates the distribution among the different types of concerns. The large majority (86.9%) felt that they, to a large or very large extent, could control their concerns.

Another recurring concern for the employees, apparent in the free text responses, was about carrying the disease to the workplace and thus infecting others, especially elderly or other vulnerable people. The respondents managed the changes caused by COVID-19 in different ways, which is illustrated in Table 3.

Table 4 shows the results of a logistic regression analysis for mental well-being (measured with the WEMWBS) below or above 44.

The results displayed in Table 4 suggest that six predictors significantly enable us to predict mental well-being (measured with the WEMWBS): perceived stress, job-satisfaction, work ability, age and education. Employees with high levels of perceived stress were more likely to have a WEMWBS score below 44 and thus having poor mental well-being (OR < 1.00). Employees aged 19–50 years and with a university degree of minimum three years of length, also had increased odds of a score below 44 (OR < 1.00). The probability of having a WEMWBS score above 44 was higher for employees who were satisfied with their job and reported higher levels of work ability (OR > 1.00).

### 3.2. The Relationship between COVID-19 Restrictions and Working Life, Family Life and Social Life

#### 3.2.1. Working Life

Around two thirds (66.8%) of the respondents experienced that the restrictions, to a large or very large extent, had affected their working life. The most influential changes were working other hours than usual, only or primarily working from home, working fewer hours, and having other tasks. Of the people that worked from home, 37.2% found it difficult to solve their work tasks satisfactory. Table 5 provides an overview of the challenges of working from home during lock-down, the main challenge being the changed communication and collaboration with colleagues.

Results from a paired samples *t*-test showed that the respondents rated all but one of the 15 items addressing work commitment, work and family life, work satisfaction and work ability significantly lower during COVID-19 (means ranging from 2.7 to 8.0) than before the outbreak (means ranging from 2.4 to 6.8) (all *p* values < 0.005).

The largest branches of industry represented in the survey were teaching, child and youth care and health services (see Table 1). To explore whether there were differences between these branches, the employees´ perceived stress, mental well-being, (during COVID-19) job satisfaction and (during COVID-19) work ability was compared. We grouped the variable ‘branches of industry’, which initially consisted of 21 categories, to four categories: teaching, child and youth care, health services and other branches. The category other branches was comprised by the remaining branches in the survey, e.g., employees in social agencies, production industry, building industry, long-term care sector, financial sector and within administration. Almost half of the respondents in this group (*n* = 268) were employees in the private sector, compared to the other three categories, which primarily were employed in the public sector.

The results from the ANOVA tests showed that employees in health services assessed their mental well-being significantly higher than employees in the category other branches (but not teaching or child and youth care) (F (3, 1324) = 5.32, *p* < 0.05). Employees in health services also assessed their work ability significantly higher than employees in child and youth, teaching and in other branches (F (3, 439) = 10.9, *p* < 0.05). The differences between the four groups of employees in terms of perceived stress and job satisfaction were not significant (*p* values > 0.05).

#### 3.2.2. Family and Social Life

A total of 69.2% of the respondents experienced that the restrictions, to a large or very large extent, had negatively affected their social life. When asked about the impact of the COVID-19 restrictions on their family life, 42.9% answered that the restrictions to a large or very large extent had affected their family life. The free text responses revealed that, as opposed to working life, more people mentioned the positive effects of the restrictions on family life, such as spending more time with their family and enjoying that in a different way than usual.

Table 6 shows the results of a logistic regression analysis for mental well-being (measured with the WEMWBS) below or above 44.

The results displayed in Table 6 suggested that five predictors were significantly associated with whether the employees had children or not: perceived stress, work ability, age and education. Employees without children under the age of 18 tended to have lower levels of perceived stress and to be older (OR < 1.00). They were also more likely to have a better work ability and a university degree of minimum 3 years of length (OR > 1.00)

#### 3.2.3. Gender Differences

As mentioned above, most of the participants in this survey were women (*n* = 1025, 77.2%). In the logistic regression analyses, we found no gender differences. Overall, however, more women than men reported that the COVID-19 restrictions negatively affected their mental well-being, family life, social life and work (see Table 7).

Furthermore, more women than men had worries because of COVID-19 (see Table 8).

## 4. Discussion

In this study, 1328 Faroese employees answered a survey about the impact of COVID-19 on psychological working environment and mental well-being during the lock-down from March to May 2020. In this period, many employees worked from home and limited their interaction with leaders and colleagues to prevent the disease from spreading. These initiatives also limited the possibility of collaborating and socializing with colleagues.

Overall, the employees´ mental well-being (according to the Warwick–Edinburgh Mental Well-Being Scale) was on an average level, with a mean score of 50.7. A score in the range 41–44 has been found to correspond with possible depression [30], so the respondents´ mean score on the WEMWBS is well above the level of depression. The results also showed that 21.8% of the respondents scored below 44, meaning that quite a large proportion of employees reported a mental well-being corresponding with depression in comparison with, e.g., the prevalence of depression in Denmark, which is around 3–4% [35]. It is worth noting, though, that the WEMWBS is not a screening tool for depression and thus not a reliable measure of prevalence. In our study, perceived stress was associated with poor mental health, which makes sense considering the interrelatedness of these constructs: high levels of stress typically have an adverse effect on the overall mental well-being and vice versa. A university degree also predicted poor mental well-being in our study, which is similar to results from a recent Spanish study that found a relationship between higher education and higher levels of depression [14]. In other studies, higher socioeconomic status has been associated with better mental well-being and health in general [36,37]. We propose two ways of interpreting this result. First, Faroese employees with a university degree are more likely to have demanding jobs, such as leading workplaces and/or teams. This task was especially challenging during lock-down and thus might have affected these employees´ mental well-being negatively. Second, the result can be interpreted in light of selection bias, because of the homogeneity of the sample. As Table 1 illustrates, the sample consisted mainly of well-educated women in their mid-thirties, which can influence the generalizability of the results. Job satisfaction and better work ability also predicted a better mental well-being.

Health care professionals rated their experience of working environment during lock-down higher compared to other professionals. In fact, employees in health care had a significantly better mental well-being than employees in the category other branches. Employees in health care also assessed their work ability significantly higher than employees in teaching, child/youth care and in other branches. These results are inconsistent with previous research that found a high prevalence of stress, anxiety, depression and other mental health difficulties among especially health care workers [9,10,11]. Our results could be explained by the nature of the changes caused by the restrictions for the different professions. Although health care professionals experienced changes in the organization of their work (e.g., working in smaller teams, longer shifts), they still maintained their core tasks, whereas teachers and professionals within teaching and child/youth care had to solve their core tasks in a different way than before, e.g., by teaching online or taking care of small children without too close physical contact. Besides suddenly finding new ways to solve the core task, the relationship between teacher and student/child, which is also a fundamental part of the core task, is compromised. The low rating of work ability could be understood in the light of these changes. Within the health care system, all non-acute treatments such as operations and outpatient treatments were postponed, and the number of COVID-19 related admissions turned out to be much lower than anticipated, which overall lessened the workload for the employees in the health care system. In fact, during the first wave, only eight patients were admitted to hospital, and no intensive care unit admissions or fatalities occurred [38]. The world-wide public support and celebration of front-line workers, especially nurses, doctors and first responders, is also likely to affect the mental well-being of health care workers. Appropriate acknowledgement can foster resilience and thus prevent mental health difficulties [39].

Although the mental health among Faroese employees was on an average level during lock-down, the respondents´ working life seemed to be worse. Two thirds of the respondents reported that the COVID-19 restrictions greatly affected their working life, which was visible in the significantly lower ratings of work commitment, work and family life, work satisfaction and work ability compared to how they usually feel about their work. These findings are consistent with previous research that found a high prevalence of work exhaustion and burnout among employees [12]. The dissatisfaction with the working environment during COVID-19 could be partly explained by the changed contact to colleagues, which employees found to be most challenging in remote working. Changed contact or no contact at all between colleagues affects the social capital negatively, compromising the possibility of forming and maintaining close workplace relations [6], and could in this study be linked to the significantly lower ratings of work commitment, work and family life, work satisfaction and work ability compared to how they usually feel about their work.

Employees experienced more positive consequences of the COVID-19 restrictions in their family life compared to their working life. Lock-down of schools and workplaces meant more time at home with family, and some employees seemed to make the most of that time by doing activities, that are more driven by pleasure than by duty and activities that have a positive effect on mental well-being. The most widely used coping mechanisms among the Faroese employees were, e.g., maintaining a normal everyday life, paying attention to the news, maintaining regular contact with family and friends; mechanisms that are in line with advice from WHO on how to cope with stress during COVID-19 [40]. The respondents more often used healthy coping mechanisms instead of less healthy coping mechanisms such as using smoking or alcohol to deal with the situation. These activities correspond with programs promoting mental health such as the ‘ABC for mental health’, where the three components of positive mental health are being active, feeling connected to other people and doing something meaningful [41]. The positive consequences or positive coping mechanisms might have served as protective factors, preventing mental well-being from deteriorating, explaining parts of the main result which was that the respondents reported an average level of mental health. Although family can be considered a protective factor to prevent mental health from deteriorating, our study also suggested that employees with children under the age of 18 tended to experience more stress and a lower work ability during COVID-19. This result is somewhat consistent with the study of Pesce and Sanna who found that having young children at home was significantly associated with mood-worsening during lock-down [19]. Balancing work and family simultaneously with home-schooling and remote working was difficult and affected the ability to perform the work-related tasks. We found that more women than men had worries because of COVID-19, which is consistent with some of the previous research [19,21,42]. The level of worrying might be understood as a reflection of the fact that the lock-down period put an extra strain on especially women. The International Labour Organization estimates that women, under normal circumstances, perform a daily average of 4 h and 25 min of unpaid care work against 1 h and 23 min for men [43]. The changes caused by COVID-19, increasing the daily time spent in unpaid work for women, is likely to be one factor contributing to the extra worrying for women in this survey.

As mentioned in the introduction, the Faroe Islands have managed to quickly gain control of three major peaks in number of infected cases since March 2020, which can be explained in several different ways. One of the measures used has been regular information from the authorities using press conferences and updated information on the official website, bringing forth an awareness among the Faroese people to change their behavior without using laws and legislation. The awareness and perception of risk has most likely contributed to behavior that minimized the spread of the virus [44]. Risk perception has in previous research been associated with the adoption of preventative health behaviors such as social distancing [45]. Another regulation of behavior comes from social control, i.e., how the surrounding society regulates our behavior [46]. In small, close knit societies, this mechanism tends to be more active, and might serve as an extra regulation to ensure that people are keeping the COVID-19 recommendations from the authorities, partly in fear of possible social consequences. A concern among the employees in this survey was the fear of being the disease carrier in the workplace and possibly infecting vulnerable people, e.g., in nursing homes. The fear of being stigmatized as a disease carrier or as infected has from other infectious diseases proven to be an important issue with implications for mental health, and the fear of stigmatization is thus an incentive to behavioral change [47,48].

One major strength of this study is that it includes employees from a broad range of professions, and not only health care professionals, as most of the previous research in the psychological effects of COVID-19 has focused on. It thus broadens the perspective concerning the effect of COVID-19 on employees. This study also has some limitations that should be taken into consideration. First, the cross-sectional research design prevents us from including the effect of time and, e.g., drawing conclusions on causal relationships. Second, this study was an online study, shared primarily through social media. This may have caused selection bias, attracting those who are active on social media and with a much higher representation of women, especially well-educated women within health care, teaching and child/youth care. This bias could affect the results, seeing that higher socio-economic status, including higher income and educational attainment, previously has been associated with preventative health behaviors [37]. The results may, therefore, not be representative for the entire population, and thus limit the generalizability.

## 5. Conclusions

In conclusion, the mental well-being of most Faroese employees was on an average level during lock-down in April and May 2020. This result may be partly linked to the effective elimination of COVID-19 in the Faroe Islands, because it shortened the lock-down period compared to other countries. Although the average mental well-being was above clinical range, a proportion of 21.8% reported a mental well-being that corresponds with depression, which is a large part compared to prevalence studies. High levels of stress predicted a poor mental well-being while high levels of job satisfaction and work ability predicted a better mental well-being.

The respondents assessed their working environment (work commitment, work and family life, work satisfaction and work ability) significantly worse during the COVID-19 outbreak than before, and employees with children tended to experience more stress and to assess their work ability during COVID-19 significantly lower than employees without children. This was more evident among women who worried more than men about the consequences of COVID-19 and felt more negatively affected by COVID-19 than men.

The influence of the lock-down on mental health has not received much attention in the public and the Faroese authorities have primarily focused on measures to contain the spread of the virus and protect the susceptible citizens, and much less on how to stay mentally healthy during the pandemic. The results from our survey highlight the importance of addressing mental health and working environment in a pandemic such as COVID-19 which may have far-reaching implications on people´s daily life. Some employees highlighted the positive effects of lock-down, e.g., spending more time with family, while others reported negative effects during lock-down. Working parents might be especially receptive of the negative effects because of the shift in domestic care obligations and the increased burden of unpaid work during COVID-19. Furthermore, short-term effects of adverse working environment, stress and worrying can have long-term implications for individual mental health and family functioning.

## Figures and Tables

**Table 1:** Characteristics of study participants in the anonymously completed self-report survey in the Faroe Islands conducted from 13 April 13 until 4 May 2020, *n* = 1328.

Participant Characteristics	
Age in years, M	34
Female, *n* (%)	1025 (77.2)
Education: University degree (min. 3 years), *n* (%)	982 (74)
Employment status: In the public sector, *n* (%)	989 (74.5)
Largest branches of industry: Teaching, *n* (%) Child and youth care, *n* (%) Health services, *n* (%) Other branches ^a^, *n* (%)	249 (18.8) 209 (15.7) 197 (14.8) 673 (50.7)
Marital status: Married/in a relationship, *n* (%)	1114 (83.9)
Children under the age of 18: yes, *n* (%)	796 (59.9)

^a^ This category is comprised by the remaining branches.

**Table 2:** Concerns caused by COVID-19 among respondents in the anonymously completed self-report survey in the Faroe Islands conducted from 13 April until 4 May 2020, *n* = 1328.

Concern Caused by COVID-19	*n* (% of Total Number of Respondents)
Well-being of the nearest relations	884 (66.6)
Future	438 (33.9)
Own well-being	336 (25.3)
Work	248 (18.7)
Economic situation	229 (17.2)
Family situation	165 (12.4)
Marriage/relationship	50 (3.8)

**Table 3:** Coping mechanisms during lock-down among respondents in the anonymously completed self-report survey in the Faroe Islands conducted from 13 April until 4 May 2020, *n* = 1328.

Coping Mechanisms during Lock-Down	N (% of Total Number of Respondents)
Maintaining a normal everyday life	946 (71.2)
Paying attention to the news	873 (65.7)
Seeking knowledge about COVID-19	764 (57.5)
Maintaining regular contact with family and friends	628 (47.3)
Eating more than usual	492 (37)
Exercising less than usual	441 (33.2)
Avoiding thinking about COVID-19	402 (30.3)
Exercising more than usual	223 (16.8)
Smoking more than usual	124 (9.3)
Drinking more alcohol than usual	119 (9)
Eating less than usual	44 (3.3)
Doing nothing to cope with the situation	21 (1.6)

**Table 4:** Data of the binary logistic regression analysis conducted to predict a Warwick–Edinburgh Mental Well-Being Scale mean score above or below 44.

Variables	OR	95% CI	*p*-Value
Perceived stress	0.51	0.44–0.59	**<0.001**
Job satisfaction (during COVID-19)	1.24	1.03–1.50	**<0.05**
Work ability (during COVID-19)	1.30	1.19–1.41	**<0.001**
Age: 19–35 years	1 (ref.)		**<0.005**
Age: 36–50 years	0.46	0.29–0.73	**<0.001**
Age: 51–71 years	0.69	0.44–1.09	0.110
Education: University degree (min. 3 years)	0.50	0.35–0.72	**<0.001**
Gender: Woman	1.40	0.96–2.07	0.079
Children: yes	1.40	0.94–2.07	0.098

Abbreviations: OR = odds ratio, CI = confidence interval, ref. = the reference category against which the other categories in the variable are compared. Significant *p*-values are in bold.

**Table 5:** Challenges of remote working during lock-down among respondents in the anonymously completed self-report survey in the Faroe Islands conducted from 13 April until 4 May 2020, *n* = 1328.

Challenges of Remote Working	N (% of Total Number of Respondents)
Contact with colleagues changed	443 (33.4)
Lack of access to tools ^a^	340 (25.6)
Lack of peace to work ^b^	291 (21.9)
Structuring the daily life for the family	284 (21.4)
Not possible to give full attention to work	280 (21.1)
Not possible to give children full attention because of work	219 (16.5)
Home-schooling	202 (15.2)
Lack of childcare	154 (11.6)
Spouse also working from home	131 (9.9)

^a^ Tools are, e.g., hardware, software and other office supplies necessary to solve the task; ^b^ The possibility to solve work-related tasks without interruptions.

**Table 6:** Data of the binary logistic regression analysis conducted to predict whether the respondents have children under the age of 18 or not.

Variables	OR	95% CI	*p*-Value
Perceived stress	0.83	0.71–0.96	**<0.05**
Job satisfaction (during COVID-19)	0.97	0.81–1.16	0.752
Work ability (during COVID-19)	1.09	1.01–1.18	**<0.05**
Age: 19–35 years	1 (ref.)		**<0.001**
Age: 36–50 years	0.17	0.12–0.25	**<0.001**
Age: 51–71 years	0.05	0.04–0.07	0.110
Education: University degree (min. 3 years)	2.03	1.46–2.81	**<0.001**
Gender: Woman	1.26	0.90–1.76	0.181
WEMWBS mean score	0.99	0.96–1.01	0.161

Abbreviations: OR = odds ratio, CI = confidence interval, ref. = the reference category against which the other categories in the variable are compared. WEMWBS = Warwick–Edinburgh Mental Well-Being Scale. Significant *p*-values are in bold.

**Table 7:** The negative effects of the COVID-19 restrictions among women and men in the anonymously completed self-report survey in the Faroe Islands conducted from 13 April until 4 May 2020, *n* = 1328.

The COVID-19 Restrictions Have, to a Large or Very Large Extent, Negatively Affected:	Women, *n* (% of Total Number of Respondents)	Men, *n* (% of Total Number of Respondents)
Mental well-being	225 (21.9)	159 (52.5)
Family Life	455 (44.5)	91 (30)
Social Life	718 (70)	50 (16.5)
Work *	730 (71.3)	46 (15.2)

* This difference was statistically significant (*p* < 0.05).

**Table 8:** Concerns caused by COVID-19 among women and men in the anonymously completed self-report survey in the Faroe Islands conducted from 13 April until 4 May 2020, *n* = 1328.

Concerns Caused by COVID-19	Women, *n* (% of Total Number of Respondents)	Men, *n* (% of Total Number of Respondents)
Well-being of the nearest relations *	725 (70.7)	159 (52.5)
Future	347 (33.9)	91 (30)
Own well-being *	286 (27.9)	50 (16.5)
Work	202 (19.7)	46 (15.2)
Economic situation	69 (22.8)	160 (15.6)
Family situation	136 (13.3)	29 (9.6)
Marriage/relationship	39 (3.8)	11 (3.6)

* These differences were statistically significant (*p* < 0.05).

## Data Availability

Data may be made available from the corresponding author upon reasonable request.
